# Major birth defects in the Brazilian side of the triple border: a population-based cross-sectional study

**DOI:** 10.1186/s13690-020-00443-w

**Published:** 2020-06-30

**Authors:** Suzana de Souza, Fernando Kenji Nampo, Cezar Rangel Pestana

**Affiliations:** 1 Latin-American Institute of Life and Natural Sciences, Federal University of Latin-American Integration, Foz do Iguassu, Parana Brazil; 2 Evidence-Based Public Health Research Group, Foz do Iguassu, Brazil; 3 Biochemistry, Toxicology and Molecular Pharmacology Research Group, Foz do Iguassu, Brazil

**Keywords:** Major birth defect, Prevalence, Risk factors, Border region

## Abstract

**Background:**

Major birth defects increase the risk of fetal death and pediatric hospitalization, which also impact on healthcare costs. Sociodemographic factors can drastically affect reproductive health and be used to discriminate the exposure to hidden risk factors. Foz do Iguassu is a Brazilian city located in the triple-border region of Brazil / Paraguay / Argentina with high rates of birth defects. However no study aimed to verify factors associated with this incidence or preventive care is reported. The current work investigated the prevalence of major birth defects and its association with maternal sociodemographic factors in Foz do Iguassu.

**Methods:**

In this population-based cross-sectional study we used data of all live births occurred in Foz do Iguassu from 2012 to 2017. The associated sociodemographic variables such as maternal age, maternal education, maternal race, country of residence, maternal parity and onset of prenatal care were analyzed. Each major birth defect was described according to absolute and relative frequencies, Kruskal-Wallis and logistic regression models were used to evaluate variables associated with selected birth defects.

**Results:**

The most prevalent major birth defects were Cleft Lip and/or Palate (9.5/10,000), gastroschisis (6.93/10,000), spina bifida (5.53/10,000), hydrocephalus (5.53/10,000), hypospadias (4.55/10,000), Down syndrome (4.23/10,000), anencephaly (2.93/10,000), anorectal atresia / stenosis (1.95/10,000), undetermined sex (1.95/10,000), esophageal atresia / stenosis with or without fistula (1.63/10,000) and limb reduction defects (1.30/10,000). Maternal age was associated with gastroschisis and Down syndrome. Only maternal education up to 7 years was statistically associated with major birth defects considering all other sociodemographic variables.

**Conclusion:**

Cleft Lip and/or Palate and Gastroschisis prevalence were higher than those found in the literature. This findings may suggest a distinct epidemiological behavior regarding major birth defects in the region. The work opens new perspectives for birth defects risk factors in the triple-border.

## Background

Birth defects are structural, functional, or metabolic disorders diagnosed at birth or in the course of life [1]. They are classified as major or minor depending on severity and represent a challenge for clinical and a public health [1–3]. The most common major birth defects involves cardiovascular and nervous systems [4, 5]. Birth defects are multifactorial and related to genetics and environmental factors, which also may vary according to sociodemographic, cultural and economic condition [6]. Sociodemographic factors may affect reproductive health by distinct exposure to risk factors as access to health services and nutrition. Identifying these factors is important to address proper preventive care [7].

In Brazil, the staff from the hospital where the delivery occurred collects data regarding pregnancy care, and maternal and newborn characteristics using a paper-based form (Declaration of Live Birth). Then, this form is sent to the Municipal Health Department, which inputs the data from all births into the web-based Information System on Live Births (SINASC). If a neonate presents a birth defect, this information is recorded into the Declaration of Live Birth and the birth defect is coded according to Chapter XVII of the International Classification of Diseases Review 10 (ICD-10). The aggregated data is available on the Brazilian government website for epidemiological assessment. Foz do Iguassu is a Brazilian city located in the triple-border region of Brazil / Paraguay / Argentina, according to government records, in the period from 2012 to 2017, Foz do Iguassu presented a birth defect rate of 11.78 / 1000 live births, roughly 50% more than that observed in the state of Paraná and Brazil (7 / 1000 live births and 8.07 / 1000 live births respectively) [8]. In addition, while about 6% of all neonatal deaths worldwide are attributed to birth defects [4], birth defects are the main cause of neonatal mortality in Foz do Iguassu, accounting for 28% of the deaths occurred within the first 28 days of life [8, 9].

Despite the high rates of birth defects in this region, the risk factors associated with its occurrence have not been studied yet. The present work aims to describe the prevalence of major birth defects and its association with maternal sociodemographic factors in Foz do Iguassu. The results may contribute to the understanding of how sociodemographic factor influence the occurrence of major birth defects in Brazil’s borderlines.

## Methods

### Study design

This is a population-based cross-sectional study.

### Settings and participants

Data regarding births in Foz do Iguassu between 2012 and 2017 was obtained from the Municipal Health Department. All live births in this period were eligible. Declaration of Live Birth forms lacking *birth defect identification* field were excluded.

### Data sources and variables

The 25 most severe major birth defects were selected to the study [10, 11]. The classification according to the affected system is presented in Table 1. The dependent variable was the presence of major birth defects and independent variables were maternal age (years); maternal education (up to 7 years of study vs more than 7 years of study); maternal race (white vs black, brown, yellow, or indigenous); country of residence (Brazil vs Paraguay or Argentina); maternal parity (primiparous vs multiparous); onset of prenatal care (first trimester vs after the first trimester).

**Table 1:** Distribution of 25 selected major birth defects according to the affected system

System	Birth defect
Central nervous system	Anencephalus
	Hydrocephalus
	Holoprosencephaly
	Spina bifida
Ear	Anophthalmia
	Anotia/microtia
Cardiovascular	Transposition of the great arteries
	Tetralogy of Fallot
	Left heart hypoplasia
	Coarctation of the aorta
Cleft Lip and/or Palate (CLP)	Cleft lip/palate/lip and/or palate
Gastrointestinal	Esophageal atresia/stenosis with or without fistula
	Small intestine atresia/stenosis
	Anorectal atresia/stenosis
Genitourinary	Hypospadias
	Undetermined sex
	Renal agenesia
	Cystic kidney
Musculoskeletal systems	Limb reduction defects
	Diaphragmatic hernia
	Omphalocele
	Gastroschisis
Chromosomal anomalies	Patau syndrome
	Edwards syndrome
	Down syndrome

### Statistical methods

Birth defects was expressed as both absolute and relative frequency (per 10,000 live births). The association between maternal age and birth defect was analyzed for each of the most prevalent major birth defects (anencephaly, spina bifida, hydrocephalus, CLP, esophageal atresia/stenosis with or without fistula, anorectal atresia/stenosis, undetermined sex, limb reduction defects, hypospadias, gastroschisis, and Down syndrome). We also pooled all birth defects to analyze the association between the other independent variables and the occurrence of birth defect.

Shapiro-Wilk test was applied to verify data distribution in each birth defect and Kruskal-Wallis test was used to verify differences between the mean maternal age of newborns with and without major defects.

Maternal education, maternal race, country of residence, maternal parity and the onset of prenatal care were described according to absolute and relative frequencies considering the distribution of newborns with and without major birth defects. To investigate the maternal sociodemographic factors associated with birth defects, a logistic regression models were proposed. Unadjusted and adjusted models were performed. The strength of association between dependent and independent variables was estimated by the Odds Ratio (OR) with 95% Confidence Interval (95% CI). Statistical analyses were conducted in Epi Info 7® and BioEstat 5.3®.

## Results

A total 30,761 births were registered in Foz do Iguassu from 2012 to 2017. 32 (0,001%) cases were excluded due to no filled birth defect identification field. Among 305 (0.99%) cases of birth defects, 140 (46%) corresponded to selected major birth defects included in this study.

CLP was the most prevalent birth defect with 9.5 / 10,000 live births (lip (*N* = 3), palate (*N* = 15), lip and palate (*N* = 11)) with mean maternal age of 26.4 ± 6.8 years. Among CLP cases, 41% (*N* = 12) presented more than one birth defect. Gastroschisis was the second most prevalent birth defect with 6.83 / 10,000 live births (*N* = 21) and a mean maternal age of 21.5 ± 4.4 years. Birth defects of nervous system (anencephaly, spina bifida, and hydrocephalus) accounted for 43 cases with a mean maternal age of 25.6 ± 9.2 years, 25.2 ± 6.5 and 24.5 ± 5.1 years, respectively. Down syndrome had 4.23 / 10,000 live births (*N* = 13) and the highest mean maternal age of 33.5 ± 7.3 years; including 3 cases with cardiovascular defect as well. Holoprosencephaly, Transposition of the great arteries, Tetralogy of Fallot, Coarctation of the aorta, Omphalocele, Patau syndrome and Edwards syndrome was no registered (Table 2).

**Table 2:** Description of the major birth defects according to the rate per 10,000 live births

Major birth defect	N	Rate/10,000
Cleft lip and/or palate	29	9.5
Gastroschisis	21	6.83
Spina bifida	17	5.53
Hydrocephalus	17	5.53
Hypospadias	14	4.55
Down syndrome	13	4.23
Anencephalus	9	2.93
Anorectal atresia/stenosis	6	1.95
Undetermined sex	6	1.95
Esophageal atresia/stenosis with or without fistula	5	1.63
Limb reduction defects	4	1.30
Left heart hypoplasia	2	0.65
Small intestine atresia/stenosis	2	0.65
Diaphragmatic hernia	2	0.65
Anophthalmia	1	0.33
Anotia/microtia	1	0.33
Renal agenesia	1	0.33
Cystic kidney	1	0.33
Total	151	44.49

No registered cases: Holoprosencephaly, transposition of the great arteries, tetralogy of Fallot, coarctation of the aorta, omphalocele, Patau syndrome, and Edwards syndrome

10 of the 140 included neonates presented more than one major birth defect

In relation to the maternal studied population, major birth defects had a higher prevalence in mothers with up to 7 years of education (30% vs 22%); white race (65.7% vs 62.9%); resident in other countries (7.9% vs. 5.5%); primiparous (42% vs 39%); onset prenatal care after the first trimester (20% vs 16%). In the logistic regression analysis, maternal education up to 7 years was the only variable associated with the major birth defects in both unadjusted and the adjusted analyzes (Unadjusted: OR = 1.46; CI 95% = 1.01–2.10; *p* = 0.0414. Adjusted: OR = 1.58; CI 95% = 1.07–2.33; *p* = 0.0213) (Table 3). Regarding maternal age, gastroschisis was statistically associated with younger mothers (21.5 ± 4.4 years) whereas Down syndrome was associated with older maternal age (33.5 ± 7.3 years) when compared to no birth defects (26.6 ± 6.5 years) (Table 4).

**Table 3:** Factors associated with major birth defects: unadjusted and adjusted odds ratio

	With major birth defect	Without major birth defect	Unadjusted			Adjusted^a^		
Variável	*N* = 140 (%)	*N* = 30,589 (%)	OR	CI 95%	***p*** value	OR	CI 95%	***p*** value
**Maternal education**								
Up to 7 years of study	41 (30)	6.739 (22)	1.46^b^	1.01–2.10	0.0414	1.58^b^	1.07–2.33	0.0213
More than 7 years of study	99 (70)	23.792 (78)	1.00	–	–	–	–	–
**Maternal race**								
White	92 (65.7)	19.118 (62.9)	1.00	–	–	–	–	–
Other	48 (34.3)	11.297 (37.1)	0.88	0.62–1.25	0.4854	0.82	0.57–1.19	0.3078
**Country of residence**								
Brazil	129 (92.1)	28.894 (94.5)	1.00	–	–	–	–	–
Others	11 (7.9)	1.695 (5.5)	1.45	0.78–2.69	0.2392	1.11	0.54–2.29	0.7611
**Maternal parity**								
Primiparous	59 (42)	11.862 (39)	1.00	–	–	–	–	–
Multiparous	81 (58)	18.596 (61)	0.87	0.62–1.22	0.4392	0.82	0.58–1.18	0.2981
**Onset of prenatal care**								
First trimester	104 (80)	24.974 (84)	1.00	–	–	–	–	–
After the first trimester	27 (20)	4.782 (16)	1.35	0.88–2.07	0.1590	1.29	0.83–1.99	0.2450

^a^Estimates adjusted for all variables in the table

^b^Statistically significant association

**Table 4:** Association between major birth defects and maternal age

	Maternal age^a^	*p* value^b^
Born without birth defect	26.6 (6.5)	
Anencephalus	25.6 (9.2)	> 0.9999
Spina bifida	25.2 (6.5)	> 0.9999
Hydrocephalus	24.5 (5.1)	> 0.9999
Cleft lip and/or palate	26.4 (6.8)	> 0.9999
Esophageal atresia/stenosis with or without fistula	20.4 (5.5)	0.2730
Anorectal atresia/stenosis	27 (5.1)	> 0.9999
Hypospadias	26.7 (5.4)	> 0.9999
Undetermined sex	25.7 (5.1)	> 0.9999
Limb reduction defects	30.3 (7.9)	> 0.9999
Gastroschisis	21.5 (4.4)	0.0022^c^
Down syndrome	33.5 (7.3)	0.0073^c^

^a^mean and standard deviation

^b^Kruskal-Wallis test

^c^statistically significant association

## Discussion

This is the first study aimed to investigate the association between sociodemographic factors and major birth defects in Brazil triple side border. The most prevalent major birth defects were CLP, gastroschisis, spina bifida, hydrocephalus, hypospadias, Down syndrome, anencephaly, anorectal atresia/stenosis, undetermined sex, esophageal atresia/stenosis with or without fistula and limb reduction defects. Maternal age was statistically positive associated with only gastroschisis and Down syndrome. Regarding other maternal sociodemographic factors, only maternal education up to 7 years was statistically associated with major birth defects.

Despite not being a major cause of mortality, CLP causes considerable morbidity and imposes a substantial financial cost for families and health system. In Brazil, 2794 surgical procedures were performed for CLP repair between 2009 and 2013, with costs above 1.507 million dollars [12]. The prevalence of CLP observed was 9.5/10,000 live births, higher than 8.23/10,000 live births found in the south Brazil. Other regions such as the northeast and southeast reported rates of 4.55/10,000 live births and 6.18/10,000 live births, respectively [13, 12]. Besides, the percentage of 41% were syndromic and associated with other cognitive or structural defects, a higher prevalence compared to approximately 30% describe in the literature [14].

Central nervous system defects accounted for 43 (31%) of total cases and have been reported as the most common outcome. This fact may reflect the complex interactions between genes and poorly understood environmental factors [15]. Literature has also shown that prenatal vitamin supplements reduce the incidence of neural tube defects [16], which emphasizes the relevance of early prenatal care particularly in the first trimester of pregnancy. Moreover, families should receive a comprehensive assistance after the diagnosis of birth defect, including better explanation about physical or mental disabilities and proper care. Besides, intrauterine surgical interventions may also be necessary after pregnancy [17].

Compared to other studies, a low number of cardiovascular birth defects (i.e. transposition of the great arteries, tetralogy of Fallot, left heart hypoplasia, or coarctation of the aorta) were observed in the present study [10, 18, 19]. These defects may not be diagnosed right after birth when data in SINASC is collected.

The association between Down syndrome and advanced maternal age is well described in the literature [20–22]. However, its prevalence was lower than reported in other studies, likely as a result of the high prevalence of young pregnant in the region. Developed countries have shown late pregnancy in women older than 35-years old increases the prevalence of Down syndrome [23].

Gastroschisis is a full-thickness defect in the abdominal wall, usually in the right side of normal umbilical cord insertion [24]. The prevalence observed in the present study was 6.83 / 10,000 live births, higher than 3.8 per 10,000 live births found in the literature [25, 26] [10, 27]. In addition, significant differences between the mean maternal age of mothers with and without gastroschisis were observed. Previous studies support the increased risks for gastroschisis with younger maternal age [28, 29]. The etiology of gastroschisis is still unclear; however, the increased risk observed among younger women suggests it may be associated with low body mass index, tobacco or drug abuse, genitourinary infections and sexually transmitted diseases [30–35].

About 60% of birth defects have unknown etiology. Genetic defects such as chromosomal disorders are more often investigated when compared to environmental factors [18]. The exposure to potential teratogens is crucial to estimate the risk associated with the social context [36, 37]. In fact, the etiological complexity of birth defects, maternal sociodemographic factors may influence adverse outcomes in embryonic development [36]. In the present study, maternal education up to 7 years was associated with major birth defects. Low maternal education may also impact on health and nutritional care with early pregnancy and exposure to teratogenic agents [37]. Another relevant social variable is access to early prenatal care including vaccination and vitamin supplementation would reduce the risk in some cases.

We also hypothesized the high incidence of some major birth defects may be related to the indiscriminated use of pesticides in the region with mutagenic and endocrine disruption properties [38]. The exposure of pregnancies to pesticides is associated with a higher chance of CLP, neural tube defects, congenital heart disease [39]. Foz do Iguassu is predominantly urban but surrounded by agricultural regions in both Brazil and Paraguay sides.

Another potential risk factor considered is the residence proximity to the Electromagnetic Fields (EMF) from one of the world largest producers of energy power lines in this area. Studies have suggested an association between exposure to EMF and leukemia, abortion, and birth defects [40–44]. We emphasize the need for additional research concerning chronic pesticides and EMFs exposure as potential harmful environmental factors.

In our study, we analyzed only records of live births based on data collected at delivery. Considering that birth defects are an important cause of abortion, the prevalence of birth defects presented in our study may be underestimated, so comparisons with other settings should be done with caution. It is important to note that the Brazilian Mortality Information System (SIM) only records fetal death that occurred after 19 weeks of gestation; thus, severe congenital defects associated with abortions within the first 19 weeks of gestation would never get registered. Also, some defects are not easily diagnosed on a physical examination of the newborn, such as cardiovascular defects, which may be underreported.

## Conclusion

The most prevalent cases of birth defects in Foz do Iguassu were CLP and gastroschisis. Moreover, the prevalence of birth defects in the region is higher than other regions. Regarding sociodemographic factors, maternal age is associated with gastroschisis and Down syndrome and education up to 7 years is determinant to the occurrence of major birth defects. Health information systems are important tools for epidemiology and data acquisition procedure should be better addressed to assure accurate diagnosis since the early stages of life.

## Data Availability

The datasets analyzed during the current study are not publicly available due to the privacy policy imposed by the Brazilian government but may be available from the corresponding author on reasonable request.

## Abbreviations

SINASC: Information System on Live Births (Sistema de Informação de Nascidos Vivos)
ICD-10: International Classification of Diseases Rev. 10
CLP: Cleft Lip and/or Palate
EMF: Electromagnetic Fields
CNS: Central nervous system
